# Elevation of circulating HLA DR^+^ CD8^+^ T-cells and correlation with chromium and cobalt concentrations 6 years after metal-on-metal hip arthroplasty: A randomized trial

**DOI:** 10.3109/17453674.2010.548028

**Published:** 2011-02-10

**Authors:** Nils P Hailer, Roman A Blaheta, Henrik Dahlstrand, André Stark

**Affiliations:** ^1^Department of Orthopedics, Institute of Surgical Sciences, Uppsala University Hospital, Uppsala, Sweden; ^2^Department of Urology, Goethe University, Frankfurt am Main, Germany; ^3^Department of Molecular Medicine and Surgery, Section of Orthopedics, Karolinska Institute, Karolinska University Hospital, Stockholm, Sweden

## Abstract

**Background and purpose:**

Following metal-on-metal hip arthroplasty (THA), immunological reactions including changes in lymphocyte populations, aseptic loosening, and lymphocytic pseudotumors occur. We hypothesized that changes in lymphocyte subpopulations would be associated with elevated metal ion concentrations.

**Methods:**

A randomized trial involving 85 patients matched for age and sex and randomized to receiving metal-on-metal (n = 41) or metal-on-polyethylene total hip arthroplasty (n = 44) was conducted. 36 patients were eligible for follow-up after mean 7 (6–8) years. Concentrations of chromium and cobalt were analyzed by high-resolution inductively coupled plasma mass spectrometry. Leukocyte subpopulations and immunoglobulins in patient blood were measured using standard laboratory methods.

**Results:**

Patients with a metal-on-metal hip had higher serum concentrations of chromium (1.05 vs. 0.36 μg/L; p < 0.001) and cobalt (0.86 vs. 0.24 μg/L; p < 0.001) than those with metal-on-polyethylene. The percentage of HLA DR^+^ CD8^+^ T-cells was higher in the metal-on-metal group (10.6 vs. 6.7%; p = 0.03) and correlated positively with chromium and cobalt concentrations in patient blood (Pearson's correlation coefficient: 0.39, p = 0.02; 0.36, p = 0.03, respectively). The percentage of B-cells was lower in the metal-on-metal group (p = 0.01). The two groups were similar with respect to immunoglobulin concentrations and Harris hip scores, and there were no radiographic signs of loosening.

**Interpretation:**

We conclude that immunological alterations appear to be associated with increased cobalt and chromium concentrations. It is tempting to speculate that HLA DR^+^ CD8^+^ T-cells are involved in the pathogenesis of allergic reactions, implant loosening, and lymphocytic pseudotumors.

Immunological phenomena—both local and systemic—that are attributed to elevated metal ion concentrations have been described after modern metal-on-metal arthroplasty: 1. Lymphocyte-mediated inflammatory reactions occur in the vicinity of metal-on-metal articulations, and polyethylene-independent osteolysis has been characterized histologically in such cases (Davies et al. 2005, Willert et al. 2005, Lazarinis et al. 2008). 2. The development of periprosthetic soft-tissue masses containing large numbers of lymphocytes has been identified as a cause of persistent pain, especially in females, after metal-on-metal hip resurfacing (Pandit et al. 2008, Toms et al. 2008). 3. At the systemic level, the induction of delayed-type hypersensitivity directed against metal ions has been observed after metal-on-metal THA (Hallab et al. 2004). 4. A decrease in the amount of circulating CD8^+^ T-cells has been described in patients with elevated metal ion levels subsequent to metal-on-metal THA, indicating further systemic immunological effects (Hart et al. 2006, 2009).

We have recently published a study of patients who were randomized to receive either a metal-on-polyethylene or a metal-on-metal bearing with a 28-mm metal head (Dahlstrand et al. 2009). Clinical parameters, radiology results, and concentrations of chromium, cobalt, nickel, and manganese were followed, and we found elevation of all metal ions after 2 years in the metal-on-metal group. In the present study, we hypothesized that immunological changes can occur as a consequence of elevated metal ion concentrations in the medium term. Specifically, in the light of previously published findings, we expected changes in subsets of CD4^+^ or CD8^+^ lymphocytes, but no gross changes in other lymphocyte subpopulations or in serum immunoglobulins.

## Patients and methods

### Study design and population

This prospective randomized study was performed in accordance with the ethical standards of the Helsinki declaration. Informed consent was obtained from all patients and the study was approved by the local ethics committee (no. 2006/958). The primary endpoints of the study were (1) the determination of concentrations of the heavy metal ions chromium, cobalt, nickel, and manganese in patient blood and (2) implant migration relative to surrounding bone, as determined by radiostereometry in 2 groups of patients that were randomized to receiving either a metal-on-metal bearing or a metal-on-polyethylene bearing. The investigation of immunological parameters was added as a secondary endpoint for this study at a later stage.

**Figure 1.**
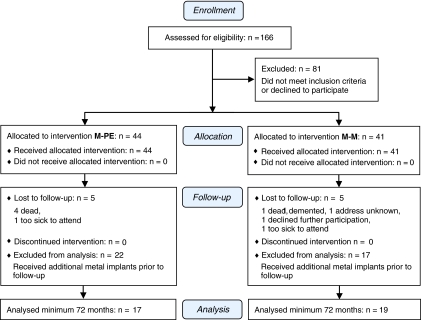
CONSORT flow chart.

166 patients, referred to the Department of Orthopedics, Karolinska Hospital, Sweden because of osteoarthritis of the hip, were eligible for study participation. Inclusion criteria were pain due to radiographically verified osteoarthritis and age between 40 and 75 years. Exclusion criteria were refusal to participate in the study, previous surgery with either osteosynthesis or joint replacement, weight over 105 kg, previous infection or surgery in the affected hip, local or general osteoporosis, intake of systemic cortical steroids for more than 3 months during the previous year, abuse of alcohol or drugs, and mental disorders including dementia. Strict application of inclusion and exclusion criteria left a cohort of 85 patients who were allocated to one of two groups according to the minimization method: 44 patients received a metal-on-polyethylene bearing and 41 patients received a metal-on-metal bearing (Figure 1). The groups were matched according to sex, smoking habits, body weight, and age. Neither the patients nor the authors involved were blinded as to the type of bearing; however, for determination of the Harris hip score, a physiotherapist blinded to the type of bearing collected the data. All patients were followed for 2 years, but 10 were subsequently lost to follow-up. Furthermore, 39 patients had to be excluded from the analysis presented here because they had received additional metal implants, rendering the measurement of metal ion concentrations meaningless. This left a study population of 36 patients, 19 in the metal-on-metal group and 17 in the metal-on-polyethylene group, who were followed for a mean of 82 (72–97) months.

### Implants

In all patients, a matte, tapered stainless steel stem (MS30; Sulzer, Winterthur, Switzerland) with a modular 28-mm head was combined with a low-profile cup. Two types of bearings were used: one consisted of a wrought high-carbon, cobalt-chromium alloy head against a cobalt-chromium liner fitted in a polyethylene cup, making it a metal-on-metal bearing (Metasul; Sulzer). The other articulation was a similar cobalt-chromium head (Protasul; Sulzer) against an all-polyethylene cup (Müller, Durasul; Sulzer). The alloy of the heads and liner consisted of a minimum of 50% cobalt, 26–30% chromium, 7% molybdenum, 1% nickel, and 1% manganese. The Metasul head is more polished than the Protasul, in order to minimize friction against the metal liner. Stem and cup were cemented with Palacos with gentamicin (Heraeus, Hanau, Germany) in all cases. All procedures were performed through a standard posterior approach by the same senior surgeon (AS) between October 1998 and January 2001.

### Clinical outcome measures and radiology

The Harris hip score (HHS) was determined preoperatively and at follow-up visits 3, 6, 12, 24, and 72 months after surgery. In all patients, follow-up included anteroposterior and lateral radiographs of the hip.

### Analysis of ion concentrations in serum

Samples of serum were collected from patients preoperatively and at 3, 6, 12, 24, and 72 months, in a standardized manner by a registered nurse using an 18-gauge intravenous cannula with the central stainless steel needle removed. The first 5 mL of blood drawn from each patient was discarded. The containers used were 10-mL polypropylene tubes with sodium heparin (Teklab Ltd., Durham, UK). One batch of cannulas and tubes was used throughout the study. All materials used to collect and store samples were chosen for their lack of metals investigated in this study.

The whole blood samples were centrifuged at 2,000 rpm for 10 min; the serum was then transferred to acid-washed polypropylene storage tubes using disposable plastic pipettes and stored at –20°C until further analysis. All samples were subsequently sent to an accredited external laboratory specialized in trace metal analysis and analyzed for concentrations of cobalt and chromium by high-resolution inductively coupled plasma mass spectrometry using a commercially available device (Element; Finnigan-MAT, Bremen, Germany). The detection limit for chromium was 0.2 μg/L (4 nMol/L) and that for cobalt was 0.05 μg/L (0.8 nMol/L). To facilitate comparisons with previous literature, ion concentrations are presented as mean concentrations in μg/L with 95% confidence intervals (CIs).

### Immunological analysis

Leukocyte subpopulations were investigated by flow cytometry on a Coulter EPICS-XL-MCL using commercially available antibodies (CD3, CD8, CD45, CD4: Tetracrome Beckman Coulter, Bromma, Sweden cat. no. 6607013; CD3, CD16/56, CD45, CD19: Tetracrome cat. no. 6607073 and Becton-Dickinson cat. no. 332779; HLA DR: Becton-Dickinson cat. no. 6604366; CD4: Becton-Dickinson cat. no. 345768; CD8: Dako cat. no. C7079). Results are given as numbers per nL, or as percentages. Immunoglobulins and IgG subclasses were analyzed by nephelometry (DADE Behrings BNll); subclasses were determined using a commercially available kit (The Binding Site Group Ltd., Birmingham, UK) and the results are expressed in g/L.

### Statistics

Before the study, a power analysis indicated that 20 patients per group would be sufficient to detect differences in metal ion concentrations (our primary endpoint) of one standard deviation with a power of 80%, given a two-tailed p-value of 0.05. The investigation of immunological parameters was not planned at the onset of the study, but was deemed to be relevant at a later stage. Thus, the power estimation was not based on this secondary endpoint. In addition, a large number of patients had to be excluded from follow-up due to the implantation of a contralateral THA, which rendered analysis of metal ion concentrations meaningless.

All variables were summarized using standard descriptive statistics such as frequencies, means, medians, and standard deviations. The distributions of metal ion concentrations were severely positively skewed; i.e., most subjects were found at the lower ends of concentration ranges, contrasted by outliers with high concentrations. By calculation of the natural logarithms of metal ion concentrations, they were transformed to normal distributions and metal ion concentrations were therefore subsequently analyzed using a linear model with cobalt or chromium concentrations after 6 years as the dependent variable, the type of bearing as the fixed factor, and preoperative cobalt or chromium concentrations as a covariate. Model residuals were normally distributed, and Cook's distances were < 1. Most immunological parameters were normally distributed, but baseline data were not available for these parameters; thus, inter-group comparisons of immunological parameters were performed using the independent t-test. Correlation analysis of logarithmically transformed metal ion concentrations with immunological parameters was performed using Pearson's correlation coefficient. The level of significance (two-tailed) was 0.05 in all analyses.

## Results

### Clinical and radiographic outcome

The two groups of patients did not differ with respect to age, sex, or BMI. Preoperatively, the HHS was 39 (CI: 32–46) in the metal-on-polyethylene group and 36 (CI: 31–41) in the metal-on-metal group. At 6 years, the HHS was 94 (CI: 89–100) in the metal-on-polyethylene group and 90 (CI: 82–98) in the metal-on-metal group.

The radiographs obtained at follow-up did not show signs of liner wear, periprosthetic osteolysis, or implant loosening in any patient.

### Concentrations of chromium and cobalt were significantly elevated 6 years after metal-on-metal arthroplasty

**Figure 2.**
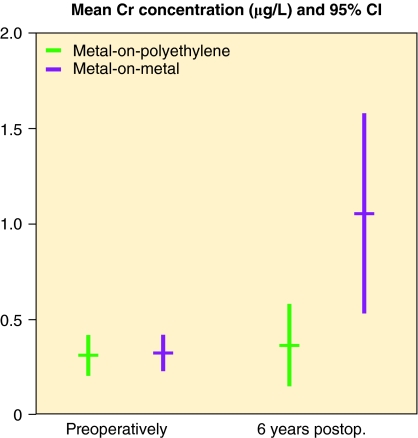
Chromium concentrations in serum prior to and 6 years after hip arthroplasty. Mean and 95% confidence intervals of chromium concentrations (in μg/L).

Preoperatively, the concentration of chromium was low and mostly below the detection limits in both groups (Figure 2). In the metal-on-polyethylene group, the mean chromium concentration was 0.31 μg/L (CI: 0.20–0.42), and it was 0.32 μg/L (CI: 0.22–0.42) in the metal-on-metal group. Postoperatively, the chromium concentrations increased substantially in the metal-on-metal group with a mean chromium concentration of 1.05 μg/L (CI: 0.53–1.6) after 6 years. In the metal-on-polyethylene group, the mean chromium concentration postoperatively showed no increase; after 6 years it was 0.36 μg/L (CI: 0.15–0.58). The chromium concentrations measured in the metal-on-metal group were substantially higher after 6 years than those measured in the metal-on-polyethylene group (p < 0.001). Baseline chromium concentrations made a minor but statistically significant contribution to the linear model (p = 0.05).

**Figure 3.**
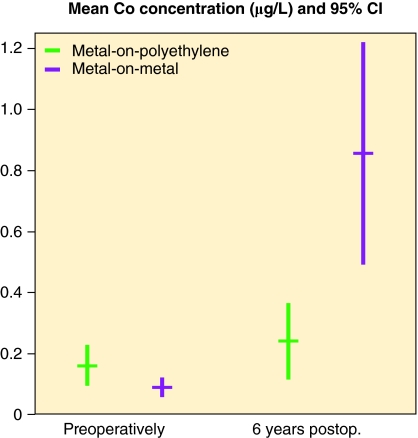
Cobalt concentrations in serum prior to and 6 years after hip arthroplasty. Mean and 95% confidence intervals of cobalt concentrations (in μg/L).

The concentration of cobalt was low preoperatively in both groups (Figure 3). The mean cobalt concentration was 0.16 μg/L (CI: 0.1–0.23) in the metal-on-polyethylene group and 0.09 μg/L (CI: 0.06–0.12) in the metal-on-metal group. 6 years after the index procedure, the mean concentration of cobalt showed a robust increase to 0.86 μg/L (CI: 0.49–1.22) in the metal-on-metal group. In the metal-on-polyethylene group, no relevant increase occurred (0.24 μg/L (CI: 0.12–0.36)). After 6 years, cobalt concentrations were higher in the metal-on-metal group than in the metal-on-polyethylene group (p < 0.001). Baseline cobalt concentrations made no statistically significant contribution to the linear model (p = 0.9).

### Number of HLA DR^+^ CD8^+^ T-cells

The number of leukocytes per nL was similar in the 2 groups of bearing types: 6.8 in the metal-on-polyethylene group and 6.4 in the metal-on-metal group (Table 1). The mean number of lymphocytes per nL was 1.9 in both groups. The concentrations of different subsets of lymphocytes (CD3^+^ T-cells, CD19^+^ B-cells, and CD16^+^/CD56^+^ NK-cells) were also similar in the 2 groups. However, a lower proportion of B-cells was found in the metal-on-metal group (9.3% vs. 12.9% in the metal-on-polyethylene group; p = 0.01).

**Table 1.** Cellular immunological parameters. Mean differences in numbers of leukocytes, lymphocytes, or lymphocyte subpopulations per nL, or percentages of lymphocytes, between the two bearing groups. Positive differences indicate higher values in the metal-on-metal group; negative differences indicate lower values in the metal-on-metal group

	Mean difference	95% confidence interval	p-value **^a^**
Leukocytes	–0.4	–1.3–0.5	0.4
Lymphocytes	0.1	–0.4–0.5	0.8
T-cells CD3	0.0	–0.4–0.4	0.9
T-cells CD3 (%)	0.8	–4.6–6.2	0.8
T-cells CD4	0.0	–0.3–0.2	0.7
T-cells CD4 (%)	–3.1	–9.5–3.3	0.3
T-cells CD4 HLA DR+	1.5	–1.3–4.2	0.3
T-cells CD8	0.1	–0.1–0.3	0.4
T-cells CD8 (%)	5.0	–1.6–11.5	0.1
T-cells CD8 HLA DR+	3.9	0.3–7.4	0.03
CD4/CD8 ratio	–1.9	–4.8–1.1	0.2
B-cells CD19	0.0	–0.1–0.0	0.2
B-cells CD19 (%)	–3.7	–6.5– -0.9	0.01
NK-cells CD16/CD56	0.1	0.0–0.2	0.1
NK-cells CD16/CD56 (%)	3.2	–1.8–8.3	0.2

**^a^** derived from independent t-test.

T-cells were further differentiated into CD4^+^ T-(helper) cells and CD8^+^ T-(cytotoxic) cells, but there were no statistically significant differences between the two bearing groups. The mean CD4/CD8 ratio was 4.7 in the metal-on-polyethylene group and 2.8 in the metal-on-polyethylene group. There were 5.1% HLA DR^+^ CD4^+^ T-cells in the metal-on-polyethylene group, as compared to 6.5% in the metal-on-metal group. A statistically significant difference between the two bearing groups was, however, found in the population of CD8^+^ T-cells positive for the HLA DR-antigen: 6.7% CD8^+^ T-cells were HLA DR^+^ in the metal-on-polyethylene group, as compared to 10.6% in the metal-on-metal group (p = 0.03).

A separate statistical analysis was done after stratifying the population for sex, but no significant differences between groups were found (data not shown).

### Correlation of HLA DR^+^ T-cells with chromium concentrations

**Figure 4.**
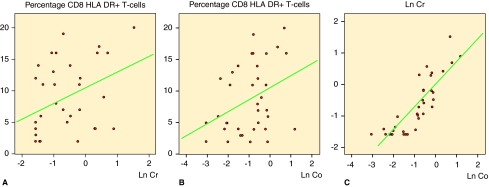
Correlation between the percentage of HLA DR^+^ CD8^+^ T-cells and metal ion concentrations after 6 years. A. A positive correlation was found between the percentage of HLA DR^+^ CD8^+^ T-cells and logarithmically transformed chromium concentrations after 6 years (Pearson's correlation coefficient: 0.39, p = 0.02). B. A positive correlation was also found between the percentage of HLA DR^+^ CD8^+^ T-cells and logarithmically transformed cobalt concentrations after 6 years (Pearson's correlation coefficient: 0.36, p = 0.03). C. Logarithmically transformed cobalt and chromium concentrations were almost linearly correlated with each other (Pearson's correlation coefficient: 0.85, p < 0.001).

Correlation analysis showed that there was a positive correlation between the percentage of HLA DR^+^ CD8^+^ T-cells and logarithmically transformed chromium and cobalt concentrations after 6 years (Figure 4 and Table 2). We also found a negative correlation between the CD4/CD8 ratio and logarithmically transformed cobalt concentrations, but no other cellular immunological parameters showed any statistically significant correlation with the concentration of either ion. Logarithmically transformed cobalt and chromium concentrations after 6 years showed a strong positive correlation with each other (Figure 4, Table 2; supplementary data).

### No effects of elevated metal ion concentrations on the humoral immune response

The concentrations of the immunoglobulins IgG (and subgroups 1–4), IgM, and IgA were similar in both groups of patients (Table 3; see supplementary data). Furthermore, there were no correlations between immunoglobulin concentrations and logarithmically transformed cobalt or chromium concentrations (Table 4; see supplementary data).

## Discussion

The main findings of our study are that: (1) serum concentrations of chromium and cobalt were elevated 6 years after metal-on-metal THA; (2) the proportion of HLA DR^+^ CD8^+^ T-cells was higher in patients who underwent metal-on-metal THA; (3) there was a positive correlation between the proportion of HLA DR^+^ CD8^+^ T-cells and both chromium and cobalt concentrations; and (4) the percentage of B-cells was reduced in the metal-on-metal group.

### Increased proportion of HLA DR^+^ CD8^+^ T-cells after metal ion exposure

Our finding that the percentage of HLA DR^+^ CD8^+^ T-cells was higher in patients with a metal-on-metal bearing has not been reported before. Against the background of the previously described reduction in the total population of CD8^+^ T-cells (Hart et al. 2006, 2009), a decrease in the number of HLA DR^+^ CD8^+^ T-cells could have been anticipated. However, in contrast to the data described by Hart et al., we found no reduction in the number of CD8^+^ T-cells in patients who underwent metal-on-metal THA. This discrepancy could be explained by differences between the various study settings. Our trial was randomized, the two groups of patients in our study were matched for age and sex, and there were no differences in BMI or time to follow-up between the two groups. In contrast, Hart et al. performed a retrospective review of a heterogeneous population; the groups investigated differed in sex distribution, age, BMI, and length of follow-up. The findings by Hart et al. are also contradicted by reports of an increased proportion of CD8^+^ T-cells and CD16^+^ NK cells in the peripheral blood of patients undergoing revision arthroplasty due to aseptic loosening (Case et al. 2000).

### Immunological reactions around arthroplasty implants

Several reports have described predominantly lymphocytic infiltrates in the neo-capsule of revised metal-on-metal THAs, consisting mainly of CD3^+^ T-cells and CD20^+^ B-cells, but also containing considerable numbers of macrophages (Davies et al. 2005, Willert et al. 2005, Witzleb et al. 2007). This type of infiltrate appears to be rather specific for metal-on-metal bearings; it is quite distinct from infiltrates that can be seen around metal-on-polyethylene THAs or in situations involving conventional deep infection. In addition, expression of interferon-γ and the presence of activated T-cells have been described in the pseudomembrane surrounding implants (Weyand et al. 1998). An immunohistochemical analysis of loose orthopedic implants with a cobalt-chromium component revealed an elevated level of HLA DR^+^ macrophages and enrichment of CD4^+^ and CD8^+^ T-cells at the bone-implant interface (al Saffar and Revell 1994).

It has also been reported that women with a metal-on-metal resurfacing arthroplasty are predisposed to development of periprosthetic pseudotumors with extensive connective tissue necrosis and mainly lymphocytic infiltration (Pandit et al. 2008). We therefore stratified our study population by sex but found no differences between the sexes. Thus, our findings cannot explain the observed predisposition of women regarding this phenomenon.

### Sensitization to heavy metal ions after THA

Sensitization to various heavy metals after THA was already described after the use of first-generation metal-on-metal arthroplasties during the 1970s, but even today it “… remains uncertain whether the loosening causes the sensitization or vice versa” (Elves et al. 1975). Even with the use of modern metal-on-metal THA, immunological reactions to heavy metal ions are known to occur, as increased lymphocytic reactivity to both cobalt and nickel has been described in patients with metal-on-metal articulations (Hallab et al. 2004). Furthermore, the lymphocytes of patients reacting to chromium more readily release interferon-γ (Hallab et al. 2008).

The mechanisms behind the immunological reactions after metal-on-metal THA are not yet understood. Macrophages at the prosthesis/tissue interface are considered to present haptenic metal ions in the context of self peptides (metal ion-altered self) as antigens in combination with MHC class II molecules, leading to T-cell priming. The mediation of metal sensitization via T-cells has been suggested by several authors: T-cell infiltrates at the implant interface suggest an association with type IV, cell-mediated sensitivity (Lalor et al. 1990), and CD3- and CD28-related signaling was observed in T-cells after exposure to cobalt/chromium-particles in vitro (Ogunwale et al. 2009).

There was a correlation between the percentage of HLA DR^+^ CD8^+^ T-cells in our study and the concentration of chromium and cobalt, although the correlation was weak. We therefore speculate that heavy metal ions released from the implant may be processed on HLA DR^+^ CD8^+^ T-cells—either like superantigens by forming and stabilizing bridges between the T-cell receptor and MHC class II molecules, or as neoantigens created by metal-protein interactions. We did not analyze cytokine release and cellular activation markers, however.

### Limitations of the study

The analysis of immunological parameters was added as a secondary endpoint after patient recruitment had been completed, and was not included in the primary study protocol. The present study, based on only 36 individuals eligible for immunological analysis, was therefore open to type-II error. At the same time, small cohorts are vulnerable to outliers, and type-I errors can therefore also occur. Approximately equal numbers were lost to follow-up or were excluded from final analysis in both study groups; we therefore believe that there was no selection bias. Taken together, our findings must be regarded as being derived from a pilot study on a small cohort of patients.

### Conclusion

Our study indicates that elevated chromium and cobalt concentrations after metal-on-metal THA correlate with immunological reactions, reflected by an elevation in HLA DR^+^ CD8^+^ T-cells and a decrease in the percentage of B-cells. We currently have no evidence that the elevation of this subtype of lymphocytes is the cause of allergic reactions, aseptic loosening, or periprosthetic pseudotumors that have been described after metal-on-metal arthroplasty. Future studies investigating a possible association between these phenomena must be performed on larger groups of patients.

## Supplementary data

Tables 2, 3 and 4 are available at our website (www.actaorthop.org), identification number 4050.

## References

[CIT0001] al Saffar N, Revell PA (1994). Interleukin-1 production by activated macrophages surrounding loosened orthopaedic implants: a potential role in osteolysis. Br J Rheumatol.

[CIT0002] Case CP, Langkamer VG, Lock RJ, Perry MJ, Palmer MR, Kemp AJ (2000). Changes in the proportions of peripheral blood lymphocytes in patients with worn implants. J Bone Joint Surg (Br).

[CIT0003] Dahlstrand H, Stark A, Anissian L, Hailer NP (2009). Elevated serum concentrations of cobalt, chromium, nickel, and manganese after metal-on-metal alloarthroplasty of the hip: A prospective randomized study. J Arthroplasty.

[CIT0004] Davies AP, Willert HG, Campbell PA, Learmonth ID, Case CP (2005). An unusual lymphocytic perivascular infiltration in tissues around contemporary metal-on-metal joint replacements. J Bone Joint Surg (Am).

[CIT0005] Elves MW, Wilson JN, Scales JT, Kemp HB (1975). Incidence of metal sensitivity in patients with total joint replacements. Br Med J.

[CIT0006] Hallab NJ, Anderson S, Caicedo M, Skipor A, Campbell P, Jacobs JJ (2004). Immune responses correlate with serum-metal in metal-on-metal hip arthroplasty. J Arthroplasty (Suppl 3).

[CIT0007] Hallab NJ, Caicedo M, Finnegan A, Jacobs JJ (2008). Th1 type lymphocyte reactivity to metals in patients with total hip arthroplasty. J Orthop Surg.

[CIT0008] Hart AJ, Hester T, Sinclair K, Powell JJ, Goodship AE, Pele L, Fersht NL, Skinner J (2006). The association between metal ions from hip resurfacing and reduced T-cell counts. J Bone Joint Surg (Br).

[CIT0009] Hart AJ, Skinner JA, Winship P, Faria N, Kulinskaya E, Webster D, Muirhead-Allwood S, Aldam CH, Anwar H, Powell JJ (2009). Circulating levels of cobalt and chromium from metal-on-metal hip replacement are associated with CD8+ T-cell lymphopenia. J Bone Joint Surg (Br).

[CIT0010] Lalor PA, Gray AB, Wright S, Railton GT, Freeman MA, Revell PA (1990). Contact sensitivity to titanium in a hip prosthesis?. Contact Dermatitis.

[CIT0011] Lazarinis S, Milbrink J, Hailer NP (2008). Avascular necrosis and subsequent femoral neck fracture 3.5 years after hip resurfacing: a highly unusual late complication in the absence of risk factors-a case report. Acta Orthop.

[CIT0012] Ogunwale B, Schmidt-Ott A, Meek RM, Brewer JM (2009). Investigating the immunologic effects of CoCr nanoparticles. Clin Orthop.

[CIT0013] Pandit H, Glyn-Jones S, McLardy-Smith P, Gundle R, Whitwell D, Gibbons CL, Ostlere S, Athanasou N, Gill HS, Murray DW (2008). Pseudotumours associated with metal-on-metal hip resurfacings. J Bone Joint Surg (Br).

[CIT0014] Toms AP, Marshall TJ, Cahir J, Darrah C, Nolan J, Donell ST, Barker T, Tucker JK (2008). MRI of early symptomatic metal-on-metal total hip arthroplasty: a retrospective review of radiological findings in 20 hips. Clin Radiol.

[CIT0015] Weyand CM, Geisler A, Brack A, Bolander ME, Goronzy JJ (1998). Oligoclonal T-cell proliferation and interferon-gamma production in periprosthetic inflammation. Lab Invest.

[CIT0016] Willert HG, Buchhorn GH, Fayyazi A, Flury R, Windler M, Koster G, Lohmann CH (2005). Metal-on-metal bearings and hypersensitivity in patients with artificial hip joints. A clinical and histomorphological study. J Bone Joint Surg (Am).

[CIT0017] Witzleb WC, Hanisch U, Kolar N, Krummenauer F, Guenther KP (2007). Neo-capsule tissue reactions in metal-on-metal hip arthroplasty. Acta Orthop.

