# Supplementary material for: Elevation of circulating HLA DR+ CD8+ T-cells and correlation with chromium and cobalt concentrations 6 years after metal-on-metal hip arthroplasty: A randomized trial
Source: Acta Orthop. 2011 Feb 10;82(1):6–12. doi: 10.3109/17453674.2010.548028 (PMC3229991; doi:10.3109/17453674.2010.548028)
Supplement: Supplementary file 1 [file ORT-1745-3674-82-006-s4050.pdf]

Supplementary data

Elevation of circulating HLA DR+ CD8+ T-cells and correlation with chromium and cobalt concentrations 6 years after metal-on-metal hip arthroplasty

A randomized trial

Nils P Hailer,[1] Roman A Blaheta[2], Henrik Dahlstrand,[3] and André Stark[3]

[1]Department of Orthopedics, Institute of Surgical Sciences, Uppsala University Hospital, Uppsala, Sweden; [2]Department of Urology, Goethe University, Frankfurt am Main, Germany; [3]Department of Molecular Medicine and Surgery, Section of Orthopedics, Karolinska Institute, Karolinska University Hospital, Stockholm, Sweden

Table 2. Correlation analysis of cellular immunological parameters and metal ion concentrations. Correlation analysis was performed using Pearson's r. There was a significant positive correlation of the percentage of HLA DR+ CD8+ T-cells with logarithmically transformed chromium and cobalt concentrations, and a highly significant positive correlation of logarithmically transformed chromium and cobalt concentrations with each other. Significant p-values highlighted in bold

		Ln Chromium	Ln Cobalt
Ln Chromium	r	1.00	0.85
	p		**<0.001**
Ln Cobalt	r	0.85	1.00
	p	**<0.001**	
Leukocytes	r	-0.01	0.04
	p	0.94	0.82
Lymphocytes	r	-0.04	0.01
	p	0.84	0.94
Tcells CD3	r	-0.05	0.01
	p	0.79	0.96
Tcells CD3 (%)	r	-0.01	0.01
	p	0.98	0.95
Tcells CD4+	r	-0.06	-0.06
	p	0.72	0.74
Tcells CD4+ (%)	r	-0.04	-0.15
	p	0.80	0.37
Tcells CD4 HLA DR+	r	0.05	0.10
	p	0.78	0.55
Tcells CD8+	r	0.05	0.12
	p	0.79	0.50
Tcells CD8+ (%)	r	0.12	0.21
	p	0.49	0.22
Tcells CD8 HLA DR+	r	0.39	0.36
	p	**0.02**	**0.03**
CD4 / CD8 –quota	r	-0.19	-0.36
	p	0.27	**0.03**
Bcells CD19+	r	-0.11	-0.21
	p	0.54	0.23
Bcells CD19+ (%)	r	-0.16	-0.29
	p	0.34	0.09
NK CD16 CD56	r	0.09	0.13
	p	0.62	0.44
NK CD16 CD56 (%)	r	0.05	0.11
	p	0.75	0.51

Table 3. Humoral immunological parameters. Mean differences in concentrations of immunoglobulins and immunoglobulin G subgroups in g/L between the two bearing groups (with 95% confidence intervals (CI), p-values derived from independent t-test). Positive differences indicate higher values in the metal-on-metal group, negative differences indicate lower values in the metal-on-metal group. No significant differences were detected

	Metal-on-metal compared with metal-on-polyethylene		
	p-value	Mean difference	95% CI
IgG	0.84	0.2	-1.7–2.1
IgA	0.79	0.1	-0.9–1.1
IgM	0.39	-0.1	-0.5–0.2
IgG1	0.55	0.3	-0.7–1.3
IgG2	0.99	0	-1.2–1.2
IgG3	0.31	-0.2	-0.5–0.2
IgG4	0.56	0.1	-0.2–0.3

Table 4. Correlation analysis of humoral immunological parameters and metal ion concentrations. Correlation analysis was performed using Pearson's r. There were no significant correlations of immunoglobulin or IgG subgroup concentrations with either ion concentration

		Ln Chromium	Ln Cobalt
IgG	r	-0.22	-0.09
	p	0.20	0.61
IgA	r	0.02	0.09
	p	0.92	0.60
IgM	r	-0.17	-0.16
	p	0.31	0.34
IgG1	r	-0.26	-0.05
	p	0.12	0.76
IgG2	r	-0.10	-0.06
	p	0.57	0.74
IgG3	r	-0.11	-0.09
	p	0.51	0.60
IgG4	r	-0.02	0.07
	p	0.89	0.70

Open Access - This article is distributed under the terms of the Creative Commons Attribution Noncommercial License which permits any noncommercial use, distribution, and reproduction in any medium, provided the source is credited.
DOI 10.3109/17453674.2010.548028
